# Pregabalin versus gabapentin in partial epilepsy: a meta-analysis of dose-response relationships

**DOI:** 10.1186/1471-2377-10-104

**Published:** 2010-11-01

**Authors:** Philippa Delahoy, Sally Thompson, Ian C Marschner

**Affiliations:** 1 Pfizer Australia, 38-42 Wharf Road, West Ryde, Sydney, NSW 2114, Australia; 2 Pfizer Limited UK, Tadworth, Surrey, KT20 7NS, UK; 3 Department of Statistics, Macquarie University, Sydney, NSW 2109, Australia

## Abstract

**Background:**

To compare the efficacy of pregabalin and gabapentin at comparable effective dose levels in patients with refractory partial epilepsy.

**Methods:**

Eight randomized placebo controlled trials investigating the efficacy of pregabalin (4 studies) and gabapentin (4 studies) over 12 weeks were identified with a systematic literature search. The endpoints of interest were "responder rate" (where response was defined as at least a 50% reduction from baseline in the number of seizures) and "change from baseline in seizure-free days over the last 28 days (SFD)". Results of all trials were analyzed using an indirect comparison approach with placebo as the common comparator. The base-case analysis used the intention-to-treat last observation carried forward method. Two sensitivity analyses were conducted among completer and responder populations.

**Results:**

The base-case analysis revealed statistically significant differences in response rate in favor of pregabalin 300 mg versus gabapentin 1200 mg (odds ratio, 1.82; 95% confidence interval, 1.02, 3.25) and pregabalin 600 mg versus gabapentin 1800 mg (odds ratio, 2.52; 95% confidence interval, 1.21, 5.27). Both sensitivity analyses supported the findings of the base-case analysis, although statistical significance was not demonstrated. All dose levels of pregabalin (150 mg to 600 mg) were more efficacious than corresponding dosages of gabapentin (900 mg to 2400 mg) in terms of SFD over the last 28 days.

**Conclusion:**

In patients with refractory partial epilepsy, pregabalin is likely to be more effective than gabapentin at comparable effective doses, based on clinical response and the number of SFD.

## Background

The primary objective of anti-epileptic therapy is to obtain complete control of seizures while minimizing the occurrence of adverse events and improving the patient's quality of life [1]. Unfortunately, a sizeable minority (15% to 30%) of patients with partial epilepsy are unsuccessfully treated with concurrent use of up to three established anti-epileptic drugs [2-4]. Such patients are candidates for adjunctive therapy with one of the many newer drugs available for epilepsy, which tend to be better tolerated than the commonly used first-line therapies and have a low propensity for drug-drug interactions [5]. While all of the new anti-epileptic drugs have satisfied the requirements of regulatory authorities by demonstrating superior efficacy relative to placebo without undue toxicity, lack of comparative, controlled clinical trial data precludes making a recommendation regarding their relative merits as adjunctive therapies.

Pregabalin and gabapentin, α2-δ ligands, are both licensed as adjunctive treatment for partial epilepsy, however head-to-head comparisons of their efficacy and safety have not been conducted. In the absence of direct, prospective, comparative studies to guide medical decision-making in the adjunctive treatment of partial epilepsy, alternative approaches are required to assess the relative value of a particular intervention versus other relevant comparators. In recent years, the role of meta-analysis has developed substantially in medical applications [6-9] and is regarded as a well accepted method of generating evidence in the context of medical decision-making and cost-effectiveness modelling [10-12]. An indirect comparison is an extension of traditional meta-analysis and includes dose-response comparisons across a range of interventions. Thus, an indirect comparison of two interventions can be made via a common comparator. In effect, an indirect comparison informs decision-makers by determining estimates of treatment effects and their statistical significance with respect to their capacity to induce an outcome of interest in the absence of direct evidence [13,14]. Therefore, we conducted an indirect comparison to assess the clinical value of adjunctive pregabalin relative to gabapentin in the management of patients with refractory partial epilepsy, with respect to the responder rate (where response was defined as a ≥50% reduction from baseline in the number of seizures) and change from baseline in seizure-free days over the last 28 days (SFD).

## Methods

### Identification and study selection

In order to identify relevant publications, a systematic literature review was performed in English on the PubMed and Thomson ISI Web of Science bibliographic databases. For example, the pregabalin search strategy on Pubmed was defined as follows: (("pregabalin"[Substance Name] OR "pregabalin"[All Fields]) AND partial[All Fields] AND ("epilepsy"[MeSH Terms] OR "epilepsy"[All Fields])) AND (Randomized Controlled Trial[ptyp] AND English[lang]). Studies were then included according to the following predetermined conditions:

*1. Study design: *Any randomized, double-blind, fixed-dose, placebo-controlled trial meeting the criteria for participants, interventions or outcomes listed below.

*2. Interventions: *Comparisons between the following interventions were of interest: low-dose pregabalin (150 mg/day) versus low-dose gabapentin (900 mg/day); mid-dose pregabalin (300 mg/day) versus mid-dose gabapentin (1200 mg/day); and high-dose pregabalin (600 mg/day) versus high-dose gabapentin (1800 mg/day). The official Summary of Product Characteristics for gabapentin as adjunctive treatment for partial epilepsy in the United States includes doses of up to 1800 mg/day, but in most other jurisdictions the recommended maximal daily dosage is up to 2400 mg/day.

*3. Study population: *Patients with partial epilepsy refractory to up to three established anti-epileptic drugs.

*4. Outcome measures: *The two outcome measures of interest were the responder rate and the change from baseline in SFD.

*5. Data extraction: *For each selected study, details were extracted on design, selection criteria, study population characteristics, interventions, outcome measures and results, which were subsequently checked by a second reviewer.

### Analysis

**Figure 1:**
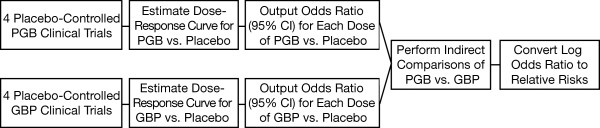
**Flow chart for analysis of responders**. CI = confidence interval; GBP = gabapentin; PGB = pregabalin

Figure 1 provides an overview of the analytical steps taken to determine responder rate estimates. Dose-response curves were estimated separately for pregabalin and for gabapentin. Dose was treated as a continuous variable, which has a logical interpretation and is an accepted approach in determining a dose-response curve [15]. Indeed, guidelines from the International Conference on Harmonisation of Technical Requirements for Registration of Pharmaceuticals for Human Use state that "study designs usually should emphasize elucidation of the dose-response function, not individual pairwise comparisons" [16]. The benefit of using a dose-response estimation technique is that information on all doses can be used to estimate a specific dose. This is particularly important for gabapentin where the estimate for the 1800 mg response is based on small patient numbers, hence information derived from previous doses are also used to inform the shape of the curve.

To compare high-dose pregabalin to high-dose gabapentin, the estimated odds ratios for each dose were compared via indirect comparisons, using placebo as the common comparator. This type of analysis also estimates the efficacy of gabapentin at the higher 2400 mg/day by extrapolating the dose response equations, and addresses the lack of clinical trial data at pregabalin 450 mg/day by interpolating the dose-response equations.

Imputed data for the base-case analysis was derived from the intention-to-treat (ITT) population and used the last observation carried forward (LOCF) method. However, concerns exist regarding whether it is appropriate to use LOCF in analyses involving progressive conditions or in situations where it may not be possible to determine whether missing data are non-random. Specifically for epilepsy, LOCF analysis yields seizure-free rates that are higher than the true clinical situation [17]. Therefore, we conducted two sensitivity analyses on the responder rate among "completer" populations to assess the robustness of the comparisons arising in the base-case analysis (i.e., pregabalin 150 mg/day versus gabapentin 900 mg/day, pregabalin 300 mg/day versus gabapentin 1200 mg/day and pregabalin 600 mg/day versus gabapentin 1800 mg/day). The first sensitivity analysis (termed the "analysis of completers") included only those patients who completed the clinical trials, and from this dataset, responders were identified. The second sensitivity analysis (termed the "analysis of responders") was akin to an ITT "missing equals failure" analysis whereby the dataset was edited in such a way that those patients who did not complete the clinical trials were classified as non-responders.

### Responder rates and logistic regression

**Figure 2:**
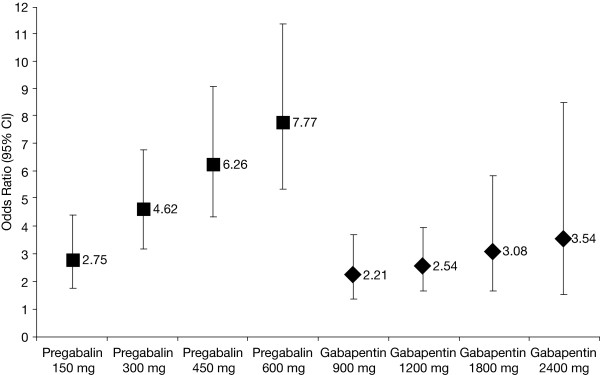
**Dose-response curves for pregabalin and gabapentin for response (base-case analysis).** The 450 mg pregabalin and 2400 mg gabapentin doses were not studied in any of the trials; the results plotted here were calculated from the dose-response equations. CI = confidence interval

**Figure 3:**
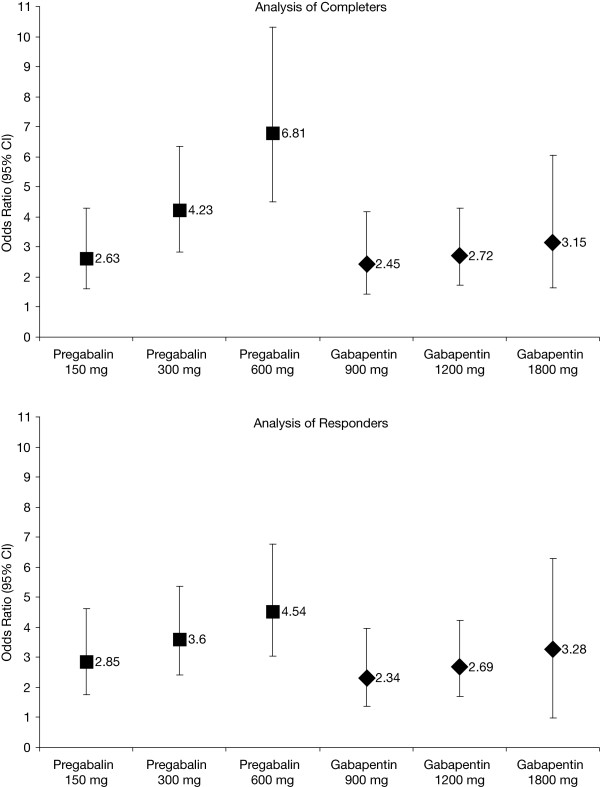
**Dose-response curves for pregabalin and gabapentin for response (sensitivity analyses)**. CI = confidence interval

Logistic regression analyses were used to model dose-response curves. For each drug, the odds ratio (vs. placebo) was modelled as a function of the drug dose. Based on exploratory analyses of the crude odds ratios (Figures 2 and 3), it was found that the log-odds ratio was a linear function of the log dosage for both drugs. Such a dose-response relationship can thus be modelled using the following logistic regression equation:

(1) log(odds)=α+βlog(dose)+γP

Here, odds is taken to mean *p*/(1-*p*) where *p *is the probability of achieving a response, while *P *is an indicator variable set at 1 for placebo and 0 for the active treatment groups.

This estimation approach enabled active doses to have a different slope and gradient than placebo, while producing both estimates within the same equation without excluding placebo. Odds ratios along with corresponding 95% confidence intervals (CIs) were reported for each dose of each drug versus placebo. That is, pregabalin doses versus placebo from pregabalin trials and gabapentin doses versus placebo from gabapentin trials.

Indirect comparisons of pregabalin and gabapentin were performed on the placebo-adjusted results of these direct comparisons. By only comparing the placebo-adjusted effects, this method of adjusted indirect comparison may preserve randomization and account for different baseline risks and other prognostic factors for participants in different trials [13].

The log odds ratio for the indirect comparison between pregabalin and gabapentin was obtained by subtracting the log odds ratios of pregabalin versus placebo, from the log odds ratios of gabapentin versus placebo.

Since the log odds ratios of pregabalin versus placebo and gabapentin versus placebo were estimated from different studies, they were statistically independent. Thus, the variance of the log odds ratios between pregabalin and gabapentin was obtained by summing the variances for the log odds ratios of each active treatment versus placebo, and 95% CIs were constructed from these variances.

Odds ratios for the indirect comparisons are presented on the original scale by taking the exponential of the estimate of the log odds ratio and bounds of the 95% Cl for the log odds ratio. For ease of interpretation odds ratios were converted to relative risk (RR) using the formula of Sutton et al [8].

(2) $$RR=\frac{OR}{1+Risk_{c}(OR-1)}$$

where Risk_c _indicates the probability of achieving a response in the placebo group [18] and Cochrane Collaboration Handbook [19].

### Change from baseline in seizure-free days over the last 28 days

This analysis used the same approach as the responder analysis except that the change from baseline in SFD is a continuous variable. That is, the same model was used as in equation (1) with the exception that the outcome variable was change from baseline in SFD. In line with good statistical practice, baseline SFD was included as a covariate. Analysis of covariance techniques were used to estimate the dose-response curve, using PROC GLM (SAS Version 8.0). Using this model, estimates of the treatment difference between active doses and placebo along with 95% CIs were obtained.

Since mean treatment differences between active doses and placebo for pregabalin and gabapentin were analyzed using separate dose response equations, the final analyses required indirect comparison between pregabalin and gabapentin for the mean difference adjusted by the results of the direct comparisons with placebo [13]. Estimates of the mean difference and variance (obtained from 95% CIs) were used in the adjusted indirect comparison. The mean difference between pregabalin and gabapentin was obtained by subtracting the mean difference of pregabalin versus placebo from the mean difference of gabapentin versus placebo.

Since the mean difference of each active treatment versus placebo was estimated from different studies, they were statistically independent. Thus, the variance of the mean difference between pregabalin and gabapentin was obtained by adding the variances for the mean difference of each active treatment versus placebo and 95% CIs were constructed from these variances.

Dose-response curves were estimated for the change from baseline in SFD for pregabalin and gabapentin separately.

## Results

Eight original research articles were identified that fulfilled the data extraction phase (Table 1). Four studies each pertained to randomized, placebo-controlled studies of pregabalin [20-23] and gabapentin [24-27]. All studies were Pfizer registration trials. The indirect comparisons in the base-case analysis involved 1911 patients, of which 674 received placebo, 807 received pregabalin, and 430 received gabapentin (Table 1). Table 1 also summarizes the number of patients completing each trial for pregabalin and gabapentin who were included in the sensitivity analyses. Most studies randomized an equal proportion of men and women, and most patients were white. In the gabapentin studies, the mean age range was 30 to 39 years. The median range of epilepsy duration across the studies was 17 to 23 years, and the median range for baseline seizure rate was ≥4 to 13 per 28 days. Overall, 51% to 68% of patients were on two concomitant anti-epileptic drugs and 0.3% to 3.0% were reported to be on three. The mean age range (36 to 41 years) in the pregabalin studies was narrower than in the gabapentin studies. The reported mean range of epilepsy duration across the studies was 23 to 27 years, and the median range for baseline seizure rate was 9 to 12 per 28 days. Relative to the gabapentin studies, the range for the proportion of patients on two concomitant anti-epileptic drugs was slightly lower in the pregabalin studies (48% to 51%) but the range for the proportion of patients on three concomitant anti-epileptic drugs was far higher (19% to 30%). Given also that around 1% of patients were on more than three concomitant anti-epileptic drugs in the pregabalin studies, it is conceivable that the pregabalin cohort had more severe refractory epilepsy than the gabapentin cohort.

**Table 1:** Summary of the multicenter, double-blind, randomized placebo-controlled trials included in the base-case and sensitivity analyses.

Individual Studies							
**Pregabalin Trials**	**Daily Dose (Titration Period)**	**No. of Patients**		**Gabapentin Trials**	**Daily Dose (Titration Period)**	**No. of Patients**	
					
		**ITT**	**Completers**			**ITT**	**Completers**

Beydoun et al 2005 [21] (Study 1008-009)	600 mg (1 week)	214	156	UK Gabapentin Study Group, 1990 [27] (Study 877-210P)	1200 mg (2 weeks)	61	54
	Placebo	98	81		Placebo	66	61

Arroyo et al 2004 [20] (Study 1008-011)	150 mg (3 days)	99	88	The US Gabapentin Study Group No. 5, 1993 [26] (Study 945-5)^d^	1200 mg (2-3 days)	101	95
	600 mg (1 week)	92	69		1800 mg (2-3 days)	54	53
	Placebo	96	84		Placebo	98	96

French et al 2003 [23] (Study 1008-034)^a,b^	150 mg	86	81	Anhut et al 1994 [24] (Study 945-6)	900 mg (2 days)	109	100
	300 mg	90	71				
	600 mg	89	61		1200 mg (2 days)	52	50
	Placebo	100	87		Placebo	109	100

Elger et al 2005 [22] (Study 1008-157)^b,c^	600 mg	137	80	Sivenius et al 1991 [25] (Study 945-9/10)	900 mg (2 days)	36	32
					1200 mg (2 days)	17	16
	Placebo	73	56		Placebo	34	30

**Aggregated Studies**							

**Treatment in Pregabalin Trials**	**Daily Dose**	**No. of Patients**		**Treatment in Gabapentin Trials**	**Daily Dose**	**No. of Patients**	
					
		**ITT**	**Completers**			**ITT**	**Completers**

Placebo		367	308	Placebo		307	287
Low-Dose Pregabalin	150 mg	185	169	Low-Dose Gabapentin	900 mg	145	132
Mid-Dose Pregabalin	300 mg	90	71	Mid-Dose Gabapentin	1200 mg	231	215
High-Dose Pregabalin	600 mg	532	366	High-Dose Gabapentin	1800 mg	54	53

All studies had a 12-week double-blind maintenance phase but the durations of the titration phases were variable.

^a^In addition to the doses listed above, for trial 1008-034 patients were also recruited into a 50 mg dose group. This dose was not found to be therapeutic and is not registered for use in Australia.

^b^No titration phase.

^c^In addition to the doses listed above, for trial 1008-157 patients were also recruited into a titrated dose arm with total daily doses ranging from 150 mg to 600 mg, depending on individual requirement. This treatment group was not comparable with the other fixed-dose treatment groups and was excluded from the analyses.

^d^In addition to the doses listed above, for trial 945-5 patients were also recruited into a 600 mg dose group. This dose was not found to be therapeutic and is not registered in Australia.

ITT = intention-to-treat.

### Logistic regression analysis of ≥50% reduction in baseline seizures: Base-case analysis (ITT LOCF)

In the base-case analysis, each dose of pregabalin was significantly different from placebo, with the magnitude of the difference increasing with dose (Table 2 and Figure 2). Odds ratios resulting from these comparisons were converted to relative risks using the Sutton formula, assuming a placebo response rate of 10%, which was the observed placebo response rate of the four studies when combined (i.e., 37 of 367 placebo patients responded). Patients with refractory epilepsy who received adjunctive high-dose pregabalin (600 mg/day) were at least four times more likely to attain a ≥50% reduction in baseline seizures patients receiving placebo (RR, 4.63; 95% CI, 3.72, 5.58) (Table 2).

**Table 2:** Odds ratios, relative risks and corresponding 95% confidence intervals (CI) for pregabalin and gabapentin versus placebo, and for pregabalin versus gabapentin in the indirect comparison using placebo as the common comparator (base-case analysis using the ITT LOCF approach)

Dose Comparison	Odds Ratio	95% CI	**Relative Risk**^**a**^	95% CI
**Pregabalin**				

150 mg vs. Placebo	2.75	1.73, 4.38	2.34	1.61, 3.27
300 mg vs. Placebo	4.62	3.16, 6.77	3.39	2.60, 4.29
450 mg vs. Placebo^b^	6.26	4.33, 9.06	4.10	3.25, 5.02
600 mg vs. Placebo	7.77	5.32, 11.34	4.63	3.72, 5.58

**Gabapentin**				

900 mg vs. Placebo	2.21	1.33, 3.68	1.97	1.29, 2.90
1200 mg vs. Placebo	2.54	1.64, 3.93	2.20	1.54, 3.04
1800 mg vs. Placebo	3.08	1.64, 5.81	2.55	1.54, 3.92
2400 mg vs. Placebo	3.54	1.48, 8.49	2.82	1.41, 4.85

**Pregabalin vs. Gabapentin**				

150 mg vs. 900 mg	1.24	0.62, 2.48	1.21	0.64, 2.16
300 mg vs. 1200 mg	1.82	1.02, 3.25	1.68	1.02, 2.65
450 mg^a ^vs. 1800 mg	2.03	0.98, 4.23	1.84	0.98, 3.21
600 mg vs. 1800 mg	2.52	1.21, 5.27	2.19	1.19, 3.69
600 mg vs. 2400 mg^a^	2.19	0.85, 5.69	1.96	0.86, 3.87

^a^These relative-risk values would change with any change in assumption regarding the placebo response rate, currently assumed to be 10%.

^b^450 mg pregabalin and 2400 mg gabapentin were not studied in any of the trials; these results were calculated from the dose-response equation.

ITT = intention-to-treat; LOCF = last observation carried forward

Similar to pregabalin, each dose of gabapentin was significantly different from placebo, with the magnitude of the difference increasing with dose (Table 2 and Figure 2). The risk for patients attaining a ≥50% reduction in baseline seizures associated with adjunctive high-dose gabapentin (2400 mg/day) was 2.82 times that of placebo. However, Figure 2 shows a greater gradient for the dose-response curve for pregabalin than for gabapentin, indicating greater incremental efficacy with higher doses of pregabalin than that attained with corresponding doses of gabapentin. Although there were overlapping 95% CIs between the pregabalin 300 mg and gabapentin 1200 mg dose levels, and between the pregabalin 600 mg and gabapentin 1800 mg dose levels, statistical significance in favor of pregabalin at these levels was indicated, and was confirmed by the indirect comparison described below [28].

Table 2 summarizes the indirect comparison for the odds ratio comparing pregabalin with gabapentin at each of their respective dose levels. Although there was a greater proportion of responders using pregabalin 150 mg than gabapentin 900 mg, this difference was not statistically significant. However the differences between pregabalin 300 mg and gabapentin 1200 mg were statistically significantly different in favor of pregabalin. There was also a statistically significant difference in favor of pregabalin 600 mg versus gabapentin 1800 mg. Patients receiving adjunctive pregabalin 300 mg and 600 mg had a 68% and 119% greater response rate than those receiving gabapentin 1200 mg and 1800 mg, respectively.

### Analysis of completers

These datasets were restricted to those patients who completed the clinical trials and from this completer patient population, responders were identified. The percentage of patients discontinuing the trials was greater at the higher doses of pregabalin than gabapentin trials. In addition, a greater percentage of placebo patient withdrew from the pregabalin than gabapentin trials.

The odds ratios and 95% CIs derived from the pregabalin and gabapentin dose-response curves among completers are summarized in Table 3. As in the base-case analysis, each dose of pregabalin and gabapentin was significantly different from placebo, and the magnitude of their effects increase with dose. The CIs around the point estimates are marginally wider, reflecting the smaller patient population. As fewer patients discontinued in the gabapentin trials, it is to be expected that the results are largely unchanged. Conversely, the magnitude of the odds ratios for pregabalin 150 mg and pregabalin 300 mg versus placebo are lower when the patients who discontinued the trial are removed from the analysis, whereas the magnitude of the odds ratio for pregabalin 600 mg versus placebo is higher. Nevertheless, Figure 3 shows that the steep gradient for the dose-response curve of pregabalin relative to gabapentin in the base-case analysis was also confirmed in the analysis of completers.

**Table 3:** Odds ratios, relative risks and corresponding 95% confidence intervals (CI) for pregabalin and gabapentin versus placebo, and for pregabalin versus gabapentin in the indirect comparison using placebo as the common comparator (sensitivity analyses)

Dose Comparison	Odds Ratio	95% CI	**Relative Risk**^**a**^	95% CI
**Analysis of Completers**				

**Pregabalin**				

150 mg vs. Placebo	2.63	1.61, 4.30	2.26	1.52, 3.23
300 mg vs. Placebo	4.23	2.82, 6.36	3.20	2.39, 4.14
600 mg vs. Placebo	6.81	4.50, 10.30	4.31	3.33, 5.34

**Gabapentin**				

900 mg vs. Placebo	2.45	1.44, 4.17	2.14	1.38, 3.17
1200 mg vs. Placebo	2.72	1.72, 4.30	2.32	1.60, 3.23
1800 mg vs. Placebo	3.15	1.64, 6.04	2.59	1.54, 4.02

**Pregabalin vs. Gabapentin**				

150 mg vs. 900 mg	1.07	0.52, 2.21	1.06	0.55, 1.97
300 mg vs. 1200 mg	1.56	0.84, 2.87	1.48	0.85, 2.42
600 mg vs. 1800 mg	2.16	1.00, 4.68^b^	1.94	1.00, 3.42

**Analysis of Responders**				

**Pregabalin**				

150 mg vs. Placebo	2.85	1.76, 4.63	2.41	1.64, 3.40
300 mg vs. Placebo	3.60	2.41, 5.37	2.86	2.11, 3.74
600 mg vs. Placebo	4.54	3.04, 6.77	3.35	2.52, 4.29

**Gabapentin**				

900 mg vs. Placebo	2.34	1.38, 3.96	2.06	1.33, 3.06
1200 mg vs. Placebo	2.69	1.71, 4.24	2.30	1.60, 3.20
1800 mg vs. Placebo	3.28	1.72, 6.28	2.67	1.60, 4.11

**Pregabalin vs. Gabapentin**				

150 mg vs. 900 mg	1.22	0.60, 2.49	1.19	0.63, 2.17
300 mg vs. 1200 mg	1.34	0.73, 2.45	1.30	0.75, 2.14
600 mg vs. 1800 mg	1.38	0.65, 2.96	1.33	0.67, 2.47

^a^These relative-risk values would change with any change in assumption regarding the placebo response rate, currently assumed to be 10%.

^b^Not significant (rounded up to 1.00).

When these results are subject to indirect comparison using placebo as the common comparator, some differences were evident between the base-case and completer analysis. The magnitude of effects in favor of pregabalin over gabapentin at all doses in the base-case analysis are only retained for the high-dose comparison (i.e., pregabalin 600 mg vs. gabapentin 1800 mg) in the completer analysis (Table 3). Relative to odds ratios in the base-case analysis, odds ratios in the analysis of completers are low for pregabalin 150 mg versus gabapentin 900 mg (OR, 1.24 vs. 1.07) and pregabalin 300 mg versus gabapentin 1200 mg (OR, 1.82 vs. 1.56). While statistical significance is lost at the margin for the pregabalin 600 mg versus gabapentin 1800 mg comparison, the odds ratios across the two analyses were in agreement and therefore appear robust (OR, 2.52 vs. 2.16). A similar finding was observed in the relative risk data, which indicated that, among completers, adjunctive pregabalin 600 mg was associated with a 94% greater probability of a ≥50% reduction in baseline seizures than gabapentin 1800 mg. While this relative risk was statistically insignificant at the margin, it is comparable to the statistically significant increased risk estimated in the base-case analysis (RR, 2.19 vs. 1.94).

### Analysis of responders

In the analysis of responders, and consistent with the base-case analysis, each dose of pregabalin and gabapentin was significantly different from placebo (Table 3). While the magnitude of the effects of both drugs increased with dose, Figure 3 shows that the gradient of the dose-response curve for pregabalin is not as steep as that observed in the base-case analysis. The gradient of the dose-response curve for gabapentin in the analysis of responders remained largely unchanged to that in the base-case analysis. Of note, the odds ratios for the pregabalin 300 mg and 600 mg dose levels were lower than those calculated in the base-case analysis, and both dose levels were accompanied by fairly high discontinuation rates (ranging from 21% to 42%). The gabapentin data were not impacted to the same extent, as fewer patients discontinued these trials (range, 2% to 12%).

When the responder data are subject to indirect comparison using placebo as the common comparator, there were no statistically significant differences between pregabalin and gabapentin at any dose level (Table 3).

### Analysis of change from baseline in seizure-free days over the last 28 days (SFD)

**Figure 4:**
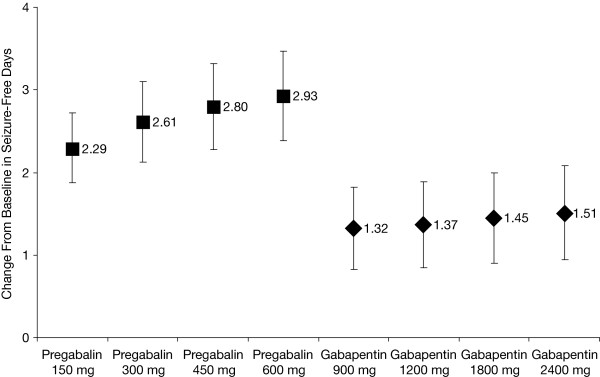
**Dose-response curves for pregabalin and gabapentin for change from baseline in seizure-free days in last 28 days**. The 450 mg pregabalin and 2400 mg gabapentin doses were not studied in any of the trials; these results were calculated from the dose-response equations.

Pregabalin and gabapentin (at all dose levels) were associated with change from baseline increases in SFD relative to placebo (Table 4). On average, patients receiving pregabalin experienced at least a 2-day increase in SFD compared with patients receiving placebo. In comparison, patients receiving gabapentin experienced at most a 1.5-day increase in SFD compared with patients receiving placebo. Figure 4 shows that the dose-response curve for pregabalin is steeper than that for gabapentin with respect to mean difference in SFD. The number of SFD did not increase appreciably with gabapentin dose.

**Table 4:** Adjusted means and mean difference between pregabalin and placebo, and gabapentin and placebo, for seizure-free days over the last 28 days

Dose	Mean Change in SFD	**S.E**.	Mean Difference Between Active and Placebo	95% CI for Mean Difference
**Pregabalin**				

150 mg	5.40	0.35	2.29	1.87, 2.72
300 mg	5.71	0.36	2.61	2.12, 3.10
450 mg^a^	5.90	0.36	2.80	2.28, 3.32
600 mg	6.03	0.36	2.93	2.38, 3.47
Placebo	3.10	0.39	-	-

**Gabapentin**				

900 mg	4.42	0.36	1.32	0.82, 1.82
1200 mg	4.48	0.37	1.37	0.85, 1.89
1800 mg	4.56	0.37	1.45	0.90, 2.00
2400 mg^a^	4.61	0.38	1.51	0.94, 2.08
Placebo	3.10	0.38	-	-

^a^Note that 450 mg pregabalin and 2400 mg gabapentin were not studied in any of the trials; these results were calculated from the dose-response equation.

CI = confidence interval; S.E. = standard error; SFD = seizure-free days over the last 28 days

The indirect comparisons shown in Table 5 suggest that for all dose comparisons, differences in the change from baseline in SFD were significantly greater with pregabalin versus gabapentin. None of the 95% CIs included zero, indicating statistical significance in favor of pregabalin. On average, patients receiving the lowest dose of pregabalin experienced a 1-day (0.97-day) increase in SFD compared with patients receiving the lowest dose of gabapentin. As the doses of both drugs increased, so did the mean difference in SFD.

**Table 5:** Mean difference in change from baseline in seizure-free days over the last 28 days for pregabalin versus gabapentin from the indirect comparison using placebo as the common comparator

Dose Comparison	Mean Difference	95% CI
150 mg vs. 900 mg	0.97	0.25, 1.69
300 mg vs. 1200 mg	1.24	0.46, 2.02
450 mg^a ^vs. 1800 mg	1.35	0.52, 2.18
600 mg vs. 1800 mg	1.48	0.62, 2.34
600 mg vs. 2400 mg^a^	1.42	0.55, 2.29

^a^450 mg pregabalin and 2400 mg gabapentin were not studied, thus, these results were calculated from the dose-response equation.

## Discussion

The objective of this analysis was to utilize existing data to compare the efficacy of pregabalin and gabapentin in order to better inform healthcare decision-makers of the relative benefits of the two treatments in the current absence of direct head-to-head comparisons. A randomized, double-blind, flexible-dose trial (ClinicalTrials.gov Identifier: NCT00537940) of pregabalin versus gabapentin as adjunctive therapy in patients with partial seizures is ongoing although the results will not be available until at least 2012.

Since all trials included in this analysis were placebo-controlled, placebo was used as the common comparator. Our base-case findings, based on ITT LOCF analysis, were that 12 weeks of mid- and high-dosage pregabalin was more efficacious than corresponding dosages of gabapentin in terms of responder rates, and that all dose levels of pregabalin were more efficacious than corresponding dosages of gabapentin in terms of SFD. A sensitivity analysis of responder rates in the completer population confirmed the benefit of mid- and high-dose pregabalin, respectively, over mid- and high-dose gabapentin, although the small sample sizes meant that the comparisons were not able to detect statistically significant between-treatment differences. Nevertheless, the data generated in the base-case analysis for the comparison between high-dose pregabalin and gabapentin was reproduced in the analysis of completers, inferring that pregabalin 600 mg is twice as likely to induce a response as gabapentin 1800 mg. In the analysis of responders, the trend in favor of pregabalin remained but statistical separation between the two drugs was not detected. One possible explanation for the observed efficacy advantage in favor of mid- and high-dose, but not low-dose, pregabalin relative to corresponding doses of gabapentin, is inherent differences in the pharmacokinetic profiles of the two drugs. Unlike pregabalin, gabapentin exhibits saturable absorption at clinically relevant dosages resulting in non-linear pharmacokinetics [29-32]. Less than dose proportional increases in gabapentin exposure are due to a saturable absorption mechanism mediated by a low capacity l-amino acid transporter found only in the upper small intestine, where gabapentin is primarily absorbed [31,33]. As the bioavailability of gabapentin decreases proportionally as the dose is increased, it is logical that statistical separation regarding responder rates is manifested at the mid- and high-dose comparisons only.

Since the two relative efficacy estimates for pregabalin and gabapentin were based on indirect comparisons, there is a greater potential for bias than if the comparisons were based on a direct prospective, randomized controlled trial comparing the two agents. Prospective, randomized controlled trials versus placebo were included in this indirect comparative analysis although it is unlikely that randomization would have held in its entirety across the studies. As a result, there was a risk that patients assigned to the different trials were not comparable regarding certain demographics and clinical characteristics. Thus, in order to minimise any bias resulting from this risk, we included in our indirect comparison only those studies that reported and were well matched regarding baseline patient characteristics (e.g., gender, age, duration of disease, baseline seizure rate). Of note, pregabalin was demonstrated to be more efficacious than gabapentin in this analysis despite one of the pregabalin studies including patients with an extremely high baseline seizure rate (21 to 25 seizures per month) [21].

Other sources of potential bias relevant to this analysis were that the included studies used slightly different designs to measure treatment effects, and that there was a relatively large time span over which the studies were conducted and published (1990 to 2005). The use of slightly different designs to measure treatment effects is not expected to impact on the conclusions of the analysis. The timing bias is more likely to be unfavourable for pregabalin. In particular there was significant growth between 1990 and 2005 in the number of alternative add-on treatments for partial epilepsy such that if patients felt they were not adequately responding to pregabalin then they were probably more likely to switch to an alternative treatment option.

All three efficacy analyses used in this study provide useful information as to the relative merits of pregabalin and gabapentin. ITT LOCF analysis will tend to overestimate response rates to pharmacotherapies in partial epilepsy trials [17], while an ITT missing equals failure analysis on the population may provide a more representative picture of real-world practice. However, an ITT missing equals failure analysis can blur the difference between two treatments when comparing a more efficacious drug associated with a high dropout rate to a less efficacious drug associated with a low dropout rate. While all three analyses showed that pregabalin was numerically more efficacious than gabapentin, the statistically significant differences in response rate for pregabalin 300 mg versus gabapentin 1200 mg and pregabalin 600 mg versus gabapentin 1800 mg derived from the base-case analysis were not achieved in the sensitivity analyses. This observation is due in part to the reduced numbers of patients in the completer analysis and the inherent variability in the data. In addition, as there were more non-completers in the placebo arm of pregabalin than gabapentin studies, the higher non-completer rate among pregabalin than gabapentin recipients may suggest that study conditions (rather than the treatments) played a role in premature patient withdrawal. One reason for the higher proportion of non-completers in the pregabalin than gabapentin trials may have been the short titration period used to achieve target pregabalin dose levels (which was one week or less in two studies and absent in the other two studies). In this regard, the sensitivity analysis may not reflect current use of pregabalin. As the pregabalin studies were conducted more recently than the gabapentin studies, another reason for the higher drop-out rates observed in the placebo arm of the pregabalin studies could be the greater availability of alternative treatment options, as discussed above.

The results generated by this analysis are consistent with findings from two meta-analyses of randomized, controlled double-blind trials, in which adjunctive pregabalin (RR 3.56; 95% CI 2.60 to 4.87; n = 1397) and adjunctive gabapentin (OR 1.93; 95% CI 1.37 to 2.71, n = 997) were significantly more likely than placebo to provide a ≥50% reduction in baseline seizures in patients with treatment-resistant partial epilepsy [34,35]. Consistent with our indirect comparison, these meta-analyses revealed that response rates increased with pregabalin dose (from 150 to 600 mg/day) and gabapentin dose (600 to 1800 mg/day), showing no evidence of an effect plateau at the doses tested [34,35]. Based partly on the conclusions reached by these systematic reviews [9,34,35], current epilepsy treatment guidelines recommend that partial epilepsy refractory to up to three anti-epileptic agents be adjunctively treated with pregabalin [5].

## Conclusions

The current analysis demonstrates that pregabalin, administered twice or three times daily for up to 12 weeks, is likely to be a more efficacious adjunctive treatment for partial epilepsy than gabapentin three times daily. Although an indirect comparison has certain inherent limitations, we believe that the performed analysis has relevancy for clinical decision-making in the treatment of partial epilepsy, in particular until the results of the direct head-to-head comparison become available.

## Competing interests

This study was funded by Pfizer Inc. Philippa Delahoy and Sally Thompson are full-time employees of Pfizer Inc. Ian Marschner was an employee of Pfizer Inc. for part of the time that this study was conducted.

## Authors' contributions

PD and IM designed the study. PD conducted the systematic review and meta-analysis, supported by IM. All authors were involved with interpreting the data statistically and clinically. All authors drafted, read and approved the final manuscript.

## Pre-publication history

The pre-publication history for this paper can be accessed here:

http://www.biomedcentral.com/1471-2377/10/104/prepub
